# The prevalence and incidence of narcolepsy in the United States: a real-world observational study using a validated narcolepsy case definition

**DOI:** 10.1093/sleepadvances/zpag043

**Published:** 2026-04-17

**Authors:** Somraj Ghosh, Veena Hoffman, Brian Calingaert, David T Plante, Lois E Krahn, Alice Cai, Mary S Anthony, Shreya Dave, Satish Rao, Stephen Crawford

**Affiliations:** Takeda Development Center Americas, Inc., Cambridge, MA, United States; RTI Health Solutions, Research Triangle Park, Durham, NC, United States; RTI Health Solutions, Research Triangle Park, Durham, NC, United States; Department of Psychiatry, University of Wisconsin School of Medicine and Public Health, Madison, WI, United States; Department of Psychiatry and Psychology and the Division of Pulmonary Medicine, Mayo Clinic College of Medicine, Phoenix, AZ, United States; Takeda Development Center Americas, Inc., Cambridge, MA, United States; RTI Health Solutions, Research Triangle Park, Durham, NC, United States; Takeda Development Center Americas, Inc., Cambridge, MA, United States; Takeda Development Center Americas, Inc., Cambridge, MA, United States; Takeda Development Center Americas, Inc., Cambridge, MA, United States

**Keywords:** case validation, narcolepsy, prevalence, incidence, United States

## Abstract

**Study Objectives:**

Narcolepsy is a chronic condition affecting the sleep/wake cycle that is challenging to diagnose. Epidemiological data from large US-focused studies remain limited, with no validated claims-based case selection definitions for narcolepsy. We estimated the prevalence/incidence of diagnosed narcolepsy (type 1 [NT1], type 2 [NT2], overall) in an insured US population using validated, claims-based narcolepsy case definitions.

**Materials and Methods:**

This observational study used HealthVerity administrative claims-linked electronic medical records to develop case definitions identifying individuals with NT1, NT2, and overall narcolepsy from diagnosis, medication, and procedure codes. Claims-based case definition performance (NT1, NT2, overall narcolepsy) was assessed using positive predictive values (PPVs). Prevalence (per 100 000 persons, on December 31, 2023) and annual incidence (per 100 000 person-years [PY], 2020 − 2023) were calculated/adjusted using PPVs for each case definition.

**Results:**

Unadjusted point prevalence among individuals with ≥12 months of continuous enrollment were 15.5/100 000 (NT1), 51.1/100 000 (NT2), and 67.0/100 000 (overall narcolepsy). Unadjusted incidence rates were stable from 2020 − 2023 for NT1 (range, 2.0 − 2.3/100 000 PY) but declined across years for NT2 (9.1 − 7.6/100 000 PY) and overall narcolepsy (11.3 − 9.6/100 000 PY). PPVs (95% confidence interval) of NT1, NT2, and overall narcolepsy were 0.81 (0.72 − 0.88), 0.84 (0.75 − 0.91), and 0.80 (0.71 − 0.87), contributing to epidemiologic estimates being reduced by ~20% after PPV adjustment. Prevalence and incidence were higher in females than males. Individuals with NT1 were ~ 4 years younger versus NT2.

**Conclusions:**

Validated definitions for narcolepsy can reduce potential misclassification. Our findings highlight a relatively low NT1 prevalence in the US. In contrast, NT2 prevalence estimates were > 3 times higher than NT1.

Statement of Significance It can be difficult to compare narcolepsy prevalence and incidence rates from different studies of administrative claims data. The values can vary considerably because of the challenges of diagnosing narcolepsy, which is often due to symptom overlap with other conditions or limited awareness among patient and healthcare practitioners. To help prevent misclassification of individuals during claims data research studies, we validated claims-based definitions of narcolepsy. To our knowledge, this is the first epidemiologic study in narcolepsy to use validated narcolepsy definitions to reduce misclassification. Our refined narcolepsy prevalence and incidence rates, estimated from an insured US population using these claims-based case definitions, may help to inform future studies.

## Introduction

Narcolepsy is a primary central disorder of hypersomnolence that disrupts the sleep/wake cycle. It is a chronic, often debilitating, condition characterized by excessive daytime sleepiness, disrupted nighttime sleep, sleep paralysis, the occurrence of hypnagogic/hypnopompic hallucinations, and cognitive symptoms. Narcolepsy may manifest as either type 1 (NT1) or type 2 (NT2), which are distinguished by the presence or absence of cataplexy, respectively and orexin deficiency (NT1) [1, 2]. The diagnosis of narcolepsy can be challenging, as individuals are often misdiagnosed due to an overlap in symptoms with other medical, neurologic, and psychiatric conditions [3–5]; there is also a lack of disease awareness and of widely used biomarkers. The formal diagnosis of narcolepsy is based on diagnostic criteria (e.g., the International Classification of Sleep Disorders - Third Edition, Text Revision [ICSD-3-TR]), which requires the presence of daily excessive daytime sleepiness for at least 3 months, and hypersomnia not explained by another sleep disorder. Diagnosis of NT1 is further defined by the presence of cataplexy with a mean sleep latency of 8 minutes or less and 2 or more sleep-onset rapid eye movement periods on a multiple sleep latency test (MSLT) of 4–5 naps or a sleep-onset rapid eye movement period on nocturnal polysomnogram and/or cerebrospinal fluid orexin A (hypocretin-1) level 110 pg/mL or less [6, 7]. In contrast, NT2 is distinguished from NT1 by the absence of cataplexy, and cerebrospinal fluid orexin A levels over 110 pg/mL [6].

Epidemiologic data on narcolepsy from large US-based studies remain limited. Existing prevalence and incidence estimates vary widely across available studies, reflecting differences in study design, data sources, and case definitions [8–13]. Additionally, diagnostic challenges may contribute to uncertainty regarding the accuracy of narcolepsy diagnoses.

In the United States, prevalence estimates arising from claims-based studies range from 38.9 to 79.4 per 100 000 individuals for narcolepsy [8, 9, 12], and incidence estimates from 5.5 to 7.7 per 100 000 person-years (PY) [8, 12]. One study using data from the US Symphony claims database reported prevalence estimates for narcolepsy between 38.9 and 44.3 per 100 000 between 2013 and 2016 [9]. Two studies using data from the US MarketScan claims database reported a higher overall prevalence of 53.3 per 100 000 persons for narcolepsy in 2019 [8], and 79.4 per 100 000 persons from 2008 to 2010 (14.0 per 100 000 persons for NT1 and 65.4 per 100 000 persons for NT2) [12]; however, the case definition used in the latter study—requiring only 1 or more claims for narcolepsy during the study period—was notably broader than those used in other studies, likely resulting in lower specificity for identifying individuals with narcolepsy. Other prevalence and incidence estimates from non–claims-based data include those from Silber et al. [13], who based estimates on data from 1960 to 1989 and reported a prevalence of 56.3 per 100 000 persons and incidence of 1.37 per 100 000 PY using Mayo Clinic criteria and 46.1 per 100 000 persons and 1.09 per 100 000 PY using ICSD criteria. In adults, the prevalence of overall narcolepsy in King County, Washington, in 2001 was 30.6 per 100 000 persons (21.8 per 100 000 for NT1). Incidence was estimated at 0.39 to 0.62 per 100 000 PY [10]. In US military personnel, the incidence of narcolepsy ranged from 14.6 to 27.3 per 100 000 PY over 2004–2013 [14]. A later study, for which estimates were based on updated ICSD-3 criteria, evaluated the prevalence of symptoms aligned with diagnoses of NT1 and NT2, reported a lower overall narcolepsy prevalence of 37.7 per 100 000 persons (12.6 per 100 000 for NT1 and 25.1 per 100 000 for NT2), with an annual incidence of overall narcolepsy of 2.6 per 100 000 PY [15].

Administrative claims data are valuable for estimating the prevalence and incidence of narcolepsy due to their large, geographically diverse populations and broad age representation. However, because claims are primarily generated for billing purposes, diagnostic codes may not reliably indicate true clinical diagnoses. Prior studies have used varying claims-based definitions with which to identify patients with narcolepsy—ranging from a single International Classification of Diseases, Ninth Revision, Clinical Modification code (347.xx) or International Classification of Diseases, Tenth Revision, Clinical Modification (ICD-10-CM) code (G47.4xx) [12, 16–18] to more complex definitions requiring multiple claims or associated procedures [9]—yet none have been formally validated. This heterogeneity of prevalence and incidence estimates highlights the need to standardize case definitions to improve precision, mitigate risks from misclassification, and generate more valid estimates.

This study addressed this gap by first validating claims-based case definitions of NT1, NT2, and overall narcolepsy, and then estimating the prevalence and annual incidence of narcolepsy using these validated case definitions. The prevalence and annual incidence estimates were adjusted to account for the performance of the case definitions and enhance the accuracy of the findings.

## Materials and Methods

### Study design

**Figure 1:**
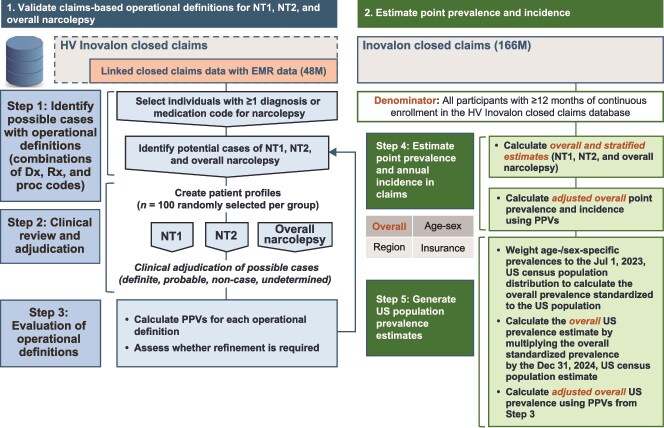
Two-part study design: Validation phase and estimation of point prevalence and incidence. Dx, diagnosis; Proc, procurement, Rx, prescription.

**Figure 2:**
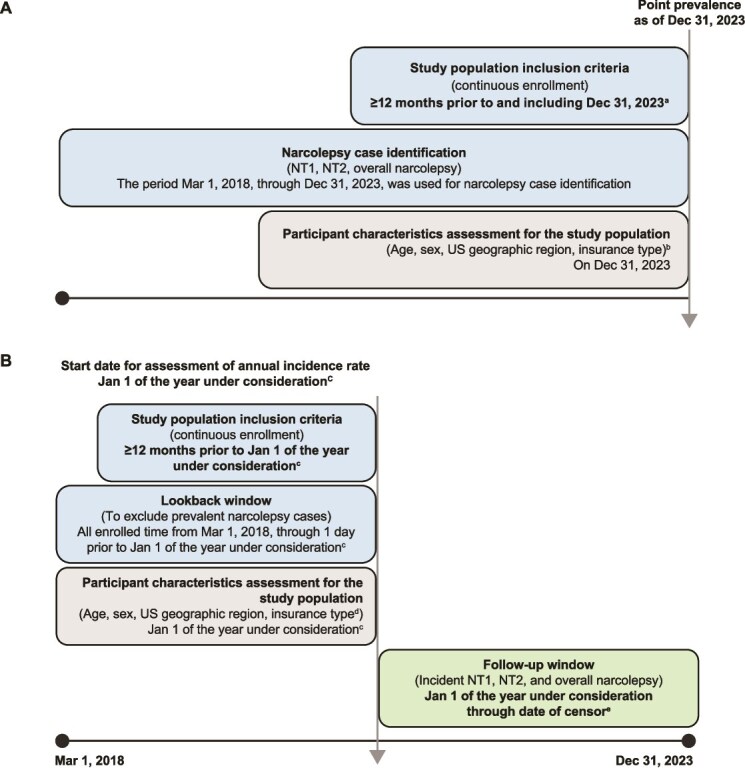
Study design to estimate (A) point prevalence of narcolepsy subtypes, and (B) annual incidence rates. ^a^The primary analysis required ≥12 months (365 days) of continuous enrollment before and including December 31, 2023. Sensitivity analyses requiring ≥24 months, ≥36 months, ≥48 months, and ≥ 60 months of continuous enrollment before and including December 31, 2023, were conducted. ^b^Age, sex, US geographic region, and insurance type were assessed on December 31, 2023, for stratification of point prevalence estimates. ^c^The year under consideration is the calendar year during which annual incidence rates were calculated. Annual incidence rates were calculated for the years 2020, 2021, 2022, and 2023. ^d^Age, sex, US geographic region, and insurance type were assessed on the date of diagnosis of NT1, NT2, and overall narcolepsy for stratification of incidence rate estimates. ^e^Incident cases of narcolepsy were identified starting on January 1 of the year under consideration until the occurrence of the earlier of (1) health plan disenrollment or (2) December 31 of the year under consideration.

This observational study employed a sequential 2-part design utilizing the HealthVerity (HV) ecosystem of databases (Fig. 1); first, to validate NT1, NT2, and overall narcolepsy operational case definitions, and second, to estimate the diagnosed point prevalence as of December 31, 2023, and annual incidence rates of NT1, NT2, and overall narcolepsy from 2020 to 2023 in the United States (Fig. 2).

The protocol was approved by RTI International’s Institutional Review Board and the study was conducted in accordance with the International Society for Pharmacoepidemiology Guidelines for Good Pharmacoepidemiology Practices [19]. Privacy rights of individuals and confidentiality of medical records were protected in accordance with all applicable laws, regulations, and guidelines. Informed consent was not required according to applicable legal requirements, as the study involved anonymized claims data, electronic medical records (EMRs), and laboratory data, and because active participation of individuals was not required. The case validation process was conducted using approaches consistent with those described in the US Food and Drug Administration’s Real-World Data: Assessing Electronic Health Records and Medical Claims Data to Support Regulatory Decision-Making for Drug and Biological Products: Guidance for Industry [20].

### Data sources

This study used secondary data from the HV ecosystem of databases. The validation of claims-based operational definitions for NT1, NT2, and overall narcolepsy was conducted among the subset of patients with Inovalon closed claims data linked to EMR data from HV Veradigm® or “Source 42.” A description of the HV Inovalon, HV Veradigm, and Source 42 data sources can be found in the Supplementary Material. Each individual was assigned a unique identification number by HealthVerity that was the same across all sources to avoid duplication of individuals in the dataset. Deidentified patient-level data from closed payer administrative medical and pharmacy claims data from HV Inovalon were used to estimate the point prevalence and the annual incidence rates of NT1, NT2, and overall narcolepsy.

### Study populations

#### Case validation

The case validation study population included patients enrolled in the HV Inovalon claims data linked to EMR data who had at least 1 medical claim with a recorded ICD-10-CM diagnosis code for NT1 and/or NT2 (G47.411, G47.421, G47.419, or G47.429). To be included, individuals were required to meet the case definition criteria defined below in the HV Inovalon claims data from March 1, 2018 (start of data availability), through December 31, 2023. Furthermore, individuals were required to have continuous medical enrollment for at least 12 months prior to and including December 31, 2023, and have at least 1 EMR record within 365 days of 1 or more narcolepsy diagnoses identified in the claims data, to maximize availability of EMR data for case adjudication. For each operational definition (NT1, NT2, and overall narcolepsy), 100 randomly selected participant profiles were built for case validation (a total of 300; see below for further details).

#### Estimation of prevalence and incidence

The study populations for point prevalence and annual incidence rate estimates were selected from individuals of any age enrolled in the HV Inovalon claims database for any length of time between March 1, 2018 and December 31, 2023. For estimation of point prevalence, participants were required to be continuously enrolled for at least 12 months prior to and including December 31, 2023. For estimation of annual incidence rates, participants were required to be continuously enrolled for at least 12 months prior to January 1 of the year under consideration. To ensure cases were incident, participants were not allowed to have received narcolepsy treatment in the year prior to diagnosis. Cases were excluded from prevalence and incidence rate calculations if the patient’s age or sex was unknown, and if their place of residence was outside of a US state.

### Case definitions

Case definitions were informed by prior studies that assessed the specificity and sensitivity of various combinations of diagnosis and procedure codes recorded in medical claims, and National Drug Codes for medications recorded in pharmacy claims [8, 21]. Cases for point prevalence and annual incidence rates for overall narcolepsy and each subtype (NT1 and NT2) were identified based on the presence of at least 2 medical claims with recorded ICD-10-CM diagnosis codes for NT1 and/or NT2 60–548 days apart, or 1 pharmacy claim with a National Drug Code for pitolisant, sodium oxybate, or mixed salt oxybate (therapies prescribed solely for the treatment of NT1 or NT2) within 365 days after the diagnosis code, or a diagnosis code within 365 days after the date of a procedure code for an MSLT (Supplementary Table S1). The definitions were designed to ensure maximum capture of true positive cases and reduce the potential for underestimating point prevalence and annual incidence. The requirement of at least 2 medical claims with a recorded diagnosis code for narcolepsy is consistent with other studies [8, 9] and reduces the potential for including patients with a single medical claim for narcolepsy that may represent a differential diagnosis coded by a provider for a procedure or test reimbursement rather than a true narcolepsy diagnosis.

NT1 and NT2 cases were identified from the overall narcolepsy cases based on the most recent diagnosis code in a participant’s claims history (ICD-10-CM G47.411 or G47.421 to identify NT1 and ICD-10-CM G47.419 or G47.429 to identify NT2). If both NT1 and NT2 were recorded on the date of the most recent diagnosis code, those cases were considered overall narcolepsy, without distinguishing between NT1 and NT2.

For point prevalence, cases were identified during all continuously enrolled time from March 1, 2018, through December 31, 2023, regardless of the minimum required enrollment. For the annual incidence rates, cases were identified starting on January 1 of the calendar year under consideration (2020, 2021, 2022, and 2023) until the occurrence of the earliest of (1) a narcolepsy diagnosis identified with the validated operational definitions; (2) health plan disenrollment; or (3) December 31 of the year under consideration. Incident cases were only counted once such that when patients met the incident case criteria in a given year, they were not included in the calculation of incidence in subsequent years.

### Validation of case definitions

Validation of potential NT1, NT2, and overall narcolepsy cases followed a multistep process that began with the random selection of 100 potential cases each for NT1, NT2, and overall narcolepsy (300 total cases). The 3 validation samples were mutually exclusive (i.e. 100 were randomly selected without replacement for the overall narcolepsy sample, then 100 each were randomly selected for the NT1 and NT2 random samples). The rationale for sampling 100 cases for overall narcolepsy and by narcolepsy subtype was based on the expected precision (width of the 95% confidence interval [CI]) in estimating positive predictive values (PPVs) to evaluate the performance of the definitions. A sample of 100 potential cases was expected to yield 95% CIs with a precision of less than ±10% for PPVs ranging from 50% to 90%.

Comprehensive deidentified patient profiles were created for all 300 cases, incorporating chronological listings of structured EMR data, available laboratory test results, and claims data, including diagnosis, procedure, and medication codes, along with the provider specialty associated with each entry. The profiles were independently adjudicated by 2 sleep specialists who then classified each case into 1 of the following categories that were developed in consultation with clinical experts: definite cases, defined as those with strong evidence supporting a narcolepsy diagnosis without data suggesting misclassification or non-narcolepsy status; probable cases, defined as those with sufficient evidence supporting a narcolepsy diagnosis without data that would reasonably increase the probability of misclassification or non-narcolepsy status; non-cases, defined as those with strong evidence supporting either misclassification of the narcolepsy category or misclassification as narcolepsy; and undetermined cases, defined as those with insufficient evidence to support the presence or absence of narcolepsy). The reviewers used their clinical judgment to determine case classification based on the presence, number, and chronological order of narcolepsy and other sleep disorder diagnosis codes, medications to treat narcolepsy, procedure claims for nocturnal polysomnography tests or MSLT, diagnosis codes for comorbidities, and the specialty of the physician associated with the claim or EMR record. Disagreement in categorization between the 2 reviewers was resolved by a third sleep specialist. If a case was classified as “definite” by 1 reviewer and “probable” by the other, it was not considered discrepant, as a probable classification could reasonably be adjudicated as definite at a different point in the diagnostic journey. The adjudicated outcomes were then used to calculate the PPVs for each operational definition. These PPVs were then applied to adjust the estimates of point prevalence and annual incidence rates. Cases classified as “undetermined” were excluded from both the numerator and denominator in the PPV calculations because the reasons for being undetermined are typically operational in nature (e.g., the participant profile reviewed did not have sufficient information available to make a case determination) and not a reflection of the claims-based case definitions.

### Statistical methods

The PPV for each case definition in the validation study was calculated as follows:


$$PPV\!=\!\frac{definite\ cases\!+\! definite/ probable\ cases\!+\! probable\ cases}{definite\ cases\!+\! definite/ probable\ cases\!+\! probable\ cases\!+\! noncases}$$

Among the population that met the prevalence study population criteria, point prevalence was calculated as the number of patients who met the case ascertainment criteria (using all available lookback time) divided by the total number of individuals in the study population. Adjusted point prevalence estimates were calculated by multiplying the unadjusted estimates by the corresponding PPVs for NT1, NT2, and overall narcolepsy. The bounds of the adjusted estimates were derived by multiplying the crude estimates by the lower and upper limits of the 95% CIs of the respective PPVs. Age- and sex-stratified point prevalence estimates calculated in the Inovalon data were weighted by the latest available age- and sex-specific US population distribution estimates (July 1, 2023) reported by the Census Bureau [22] to calculate an overall age- and sex-adjusted prevalence. To obtain a contemporary count on prevalent cases in the United States, the adjusted estimates expressed as per 100 000 individuals were factored/multiplied to the December 31, 2024, US Census population. To assess the impact of longer lookback periods on point prevalence estimates, sensitivity analyses were conducted using minimum continuous enrollment requirements of 24, 36, 48, and 60 months prior to and including December 31, 2023.

Annual incidence rates were calculated as the number of incident cases within the year under consideration divided by the amount of person-time contributed by all eligible participants within that year. Adjusted point prevalence and annual incidence estimates were calculated by multiplying the unadjusted estimates by the corresponding PPVs for NT1, NT2, and overall narcolepsy. The bounds of the adjusted estimates were derived by multiplying the unadjusted estimates by the lower and upper limits of the 95% CIs of the respective PPVs.

Prevalence and incidence were calculated overall and stratified by age category and sex and reported per 100 000 persons and 100 000 PY, respectively. Demographic characteristics are reported for the 2023 prevalence study population and the 2023 annual incidence rate population and are expressed using descriptive statistics, with means and standard deviations (SDs) or medians and interquartile ranges for continuous variables, and frequency distributions and percentages for categorial variables. All analyses for this study were conducted using SAS statistical software, version 9.4 or later (SAS Institute, Cary, NC).

## Results

### Validation of NT1, NT2, and overall narcolepsy case definitions

The starting population comprised 156 077 patients with at least 1 medical claim for narcolepsy. Of these, 84 441 met the claims-based operational definition for overall narcolepsy, and 23 239 met both the medical enrollment (≥12 months through December 31, 2023) and EMR encounter (≥1 EMR record within ±365 days of a claims-based narcolepsy diagnosis) criteria. Among these, 5148 met the NT1 definition, 17 963 met the NT2 definition, and 128 met the narcolepsy definition but the type could not be determined. From these subgroups, 100 patients were randomly selected for validation of each operational definition. The clinician adjudication results and PPVs for claims-based NT1, NT2, and overall narcolepsy case definitions are shown in Table 1. PPVs (95% CI) of claims-based definitions for NT1, NT2, and overall narcolepsy were 0.81 (0.72–0.88), 0.84 (0.75–0.91), and 0.80 (0.71–0.87), respectively.

**Table 1:** Estimated PPVs of claims-based operational definitions for NT1, NT2, and overall narcolepsy

**Narcolepsy operational definition** ^*^	**Reviewer case status decision**					**PPV (95% CI)**
	**Definite** ^**†**^	**Definite/probable^‡^**	**Probable** ^**§**^	**Non-case**	**Undetermined**	
NT1, *n*	26	31	24	19	0	0.81 (0.72–0.88)
NT2, *n*	38	30	16	16	0	0.84 (0.75–0.91)
Overall narcolepsy, *n*	9	21	49	20	1	0.80 (0.71–0.87)

^*^Claims-based operational definitions of NT1, NT2, and overall narcolepsy for validation are described in the Supplementary Material.

^†^Definite cases made by physician reviewer consensus.

^‡^Cases that 1 reviewer classified as definite, and the other reviewer classified as probable.

^§^Probable cases made by physician reviewer consensus.

Abbreviations: CI, confidence interval; NT1, narcolepsy type 1; NT2, narcolepsy type 2; PPV, positive predictive value.

### Unadjusted and PPV-adjusted point prevalence of narcolepsy

A total of 70 548 164 individuals met the enrollment inclusion criteria for estimation of point prevalence. The mean (SD) age was 35.2 (20.7) years, and more than half of the participants were female (53.2%). The study population included participants with diverse types of insurance coverage, with the majority covered by commercial health plans (44.5%) or Medicaid (49.0%), followed by Medicare Advantage (4.9%) (Supplementary Table S2). Compared with the denominator population, the narcolepsy case population had a higher proportion of females versus males, and a greater representation of adults aged 25–54 years (Supplementary Table S2).

The unadjusted point prevalences on December 31, 2023, for NT1, NT2, and overall narcolepsy among individuals with at least 12 months of continuous enrollment using all available lookback data were 15.5, 51.1, and 67.0 per 100 000 persons, and the PPV-adjusted point prevalences (95% CI) for NT1, NT2, and overall narcolepsy were 12.6 (11.2–13.6), 42.9 (38.3–46.5), and 53.6 (47.6–58.3) per 100 000 persons, respectively (Table 2). Sensitivity analyses capturing the prevalence for individuals with ≥24 to ≥60 months of continuous enrollment (i.e. minimum lookback) showed that both unadjusted and PPV-adjusted point prevalence increased with the longer lengths of minimum required lookback (Table 2).

**Table 2:** Unadjusted and PPV-adjusted point prevalence per 100 000 by lookback period

**Continuous enrollment, months** ^*^	**Denominator**	**NT1**		**NT2**		**Overall narcolepsy**	
		**Unadjusted**	**Adjusted (95% CI)**	**Unadjusted**	**Adjusted (95% CI)**	**Unadjusted**	**Adjusted (95% CI)**
**Primary analysis**							
** ≥12**	**70 548 164**	**15.5**	**12.6 (11.2–13.6)**	**51.1**	**42.9 (38.3–46.5)**	**67.0**	**53.6 (47.6–58.3)**
Sensitivity analyses							
≥24	58 850 118	16.5	13.4 (11.9–14.5)	55.0	46.2 (41.3–50.1)	72.0	57.6 (51.1–62.6)
≥36	49 484 728	17.3	14.0 (12.5–15.2)	58.0	48.7 (43.5–52.8)	75.8	60.6 (53.8–65.9)
≥48	40 547 931	18.2	14.7 (13.1–16.0)	61.5	51.7 (46.1–56.0)	80.2	64.2 (56.9–69.8)
≥60	31 446 909	19.1	15.5 (13.8–16.8)	65.3	54.9 (49.0–59.4)	84.9	67.9 (60.3–73.9)

^*^Continuous enrollment from December 31, 2023, with lookback periods from ≥12 to ≥60 months. Bold signifies the primary analysis. Bold signifies the primary analysis.

Abbreviations: CI, confidence interval; NT1, narcolepsy type 1; NT2, narcolepsy type 2; PPV, positive predictive value.

**Figure 3:**
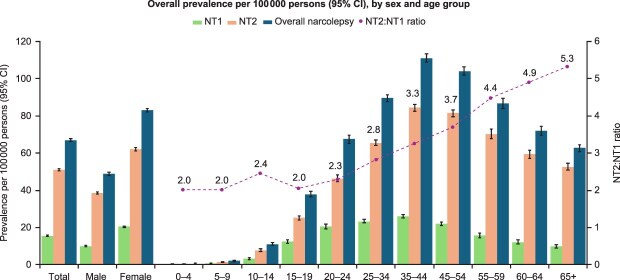
Point prevalence rates by sex and age as of December 31, 2023. Primary analysis includes patients with ≥12 months of continuous enrollment before and including December 31, 2023. Bars show prevalence per 100 000 persons; data points show NT2:NT1 ratio.

Point prevalence of NT1 and NT2 was 2.0- and 1.6-fold higher among females than males, respectively (Fig. 3). There were also observed differences in NT1 and NT2 prevalence by age. Individuals with NT1 were ~ 4 years younger, on average, than those with NT2 (mean [SD] 35.8 [15.4] vs 40.1 [15.9] years). The point prevalence of NT1 was highest among 25- to 34-year-olds and 35- to 44-year-olds, whereas the point prevalence of NT2 was highest among 35- to 44-year-olds and 45- to 54-year-olds (Fig. 3). Furthermore, the ratio of NT2:NT1 prevalence increased with age, from 2.0- to 2.4-fold higher among those under the age of 20 years, 3.7-fold higher in those aged 45–54 years old, and 5.3-fold higher among those 65 years and older.

The estimated prevalence of narcolepsy in the December 31, 2024, US population was 50 148 individuals for NT1, 176 029 individuals for NT2, and 227 541 individuals for overall narcolepsy, equating to 15.5, 51.1, and 67.0 per 100 000 persons, respectively [23]. PPV-adjusted estimates were 40 620 individuals for NT1, 147 864 individuals for NT2, and 182 033 individuals for overall narcolepsy, equating to 12.6, 42.9, and 53.6 per 100 000 persons. Prevalence estimates increased with each successive sensitivity analysis (Table 2).

### Unadjusted and PPV-adjusted annual incidence rate of narcolepsy

A total of 62 809 285 individuals met the inclusion criteria for estimation of the annual incidence rate in 2023. The mean (SD) age was 35.3 (20.9) years and 53.4% were female. As with the prevalence population, the incidence population included diverse types of insurance coverage with the majority covered by commercial (43.1%) and Medicaid (50.5%), and a smaller proportion covered by Medicare Advantage (4.8%) (Supplementary Table S2). Compared with the denominator population, the narcolepsy case population had a higher proportion of females versus males, and a greater representation of adults aged 25–54 years (Supplementary Table S2).

The unadjusted and adjusted annual incidence rates of NT1, NT2, and overall narcolepsy between 2020 and 2023 are shown in Supplementary Table S3. From 2020 to 2023, unadjusted annual incidence rates per 100 000 person-years ranged from 2.0 to 2.3 for NT1, 7.6 to 9.1 for NT2, and 9.6 to 11.3 for overall narcolepsy. After adjusting for PPVs, the corresponding estimates (95% CIs) ranged from 1.6 (1.4–1.8) to 1.9 (1.7–2.0) for NT1, 6.4 (5.7–6.9) to 7.6 (6.8–8.3) for NT2, and 7.7 (6.8–8.4) to 9.0 (8.0–9.8) for overall narcolepsy. Although the annual incidence rate for NT1 remained stable over time, the estimates for NT2 and overall narcolepsy showed a consistent decline from 2020 to 2023. The unadjusted annual incidence rate in 2023 was higher in females compared with males and incidence rates were highest for both NT1 and NT2 among adults 25–34 and 35–44 years of age, respectively (Supplementary Fig. S1).

## Discussion

Comparing narcolepsy prevalence across studies is challenging due to substantial variability in estimates, largely driven by heterogeneity in case ascertainment methodologies. When using administrative claims data, applying a carefully constructed case definition is critical, as the potential for misclassification is well recognized in the narcolepsy space [24]. Currently, there are no existing validated administrative claims-based algorithms to accurately specify narcolepsy cases. To address this gap, we evaluated the performance of claims-based case definitions (algorithms) of NT1, NT2, and overall narcolepsy with clinician adjudication of deidentified participant profiles for a randomly selected sample of possible cases prior to estimating prevalence and incidence of these conditions.

The calculated PPVs for NT1, NT2, and overall narcolepsy of 0.81, 0.84, and 0.80, respectively, suggest that the operational definitions perform reasonably well; however, given that adjudication was based on holistic participant profiles without access to sleep test results (e.g., MSLT, nocturnal polysomnography) from clinical notes, some degree of misclassification likely persists. As of December 31, 2023, the sex- and age-adjusted (to US population) point prevalences for NT1, NT2, and overall narcolepsy among individuals with at least 12 months of lookback were 15.5, 51.1, and 67.0 per 100 000 persons, respectively, while the PPV-adjusted point prevalences were approximately 20% lower (12.6, 42.9, and 53.6 per 100 000, respectively). When using a longer minimum lookback period of 36 months, the PPV-adjusted point prevalences were higher (14.0, 48.7, and 60.6 per 100 000, respectively).

Although few US-based health claims database studies have evaluated narcolepsy prevalence and incidence, we found that our PPV-adjusted estimates were consistent with previous reports. Abioye et al. reported similar prevalence estimates of 10.7 per 100 000 persons for NT1, 41.7 per 100 000 for NT2, and 53.3 per 100 000 for overall narcolepsy in 2019 using data from the IBM US MarketScan database [8]. They defined prevalent cases as individuals with at least 2 medical claims with recorded diagnosis codes for the same type of central disorder of hypersomnolence occurring at least 60 days but 12 months or less apart [8], which was slightly more stringent than the validated definition used in the current study, allowing for a longer gap between diagnosis codes for narcolepsy (occurring ≥60 days but ≤548 days apart) and additionally included patients with 1 medical claim with a diagnosis code for narcolepsy and either a claim with a narcolepsy-specific medication or MSLT procedure within 12 months.

Acquavella et al. reported a lower prevalence of 44.3 per 100 000 persons for overall narcolepsy in 2016 [9], reflecting utilization of a more stringent case definition. Cases were required to have at least 2 claims within 6 months including a diagnosis of narcolepsy, or 1 narcolepsy claim and an MSLT within the prior 6 months. Both claims had to occur within a 2-calendar-year period. Thus, these estimates of prevalence excluded cases not receiving active medical care over the specified period.

In contrast, a study by Scheer et al. using the US Truven Health MarketScan Commercial Claims and Encounters Database reported an overall narcolepsy prevalence of 79.4 per 100 000 persons during 2008–2010 [12], which is substantially higher than estimates from Abioye et al. and Acquavella et al. but closer to our unadjusted prevalence estimate of 67.0 per 100 000 persons. Importantly, the narcolepsy case definition used by Scheer et al. required only 1 or more diagnosis codes, resulting in a more inclusive approach that likely contributed to their higher estimates. Given the structure of claims databases, using a case definition based on a single claim—especially for chronic conditions—can lead to misclassification by capturing both suspected and confirmed cases [21]. The use of clinician-adjudicated claims-based case definitions in our study quantified the potential misclassification within NT1, NT2, or overall narcolepsy that, when factored in, provided more robust and plausible prevalence estimates and incidence estimates. Misclassification could be further explored through an expansion of the existing validation approach to evaluate a case definition consisting of a single narcolepsy diagnosis code, which would likely result in substantially lower PPV estimates than observed in this study.

The validated case definitions were also applied to assess trends in annual incidence rates. We found that PPV-adjusted annual incidence rates for NT1 were stable over time, ranging from 1.6 to 1.9 per 100 000 person-years between 2020 and 2023. However, the incidence rates for NT2 and overall narcolepsy decreased annually. The reason for the observed trend remains unclear, especially given the growing awareness and diagnosis of NT2 over time. One possible explanation is more limited available lookback time in earlier years (data available from March 2018) to identify prevalent cases for exclusion from consideration as an incident case. Another explanation could relate to improved nomenclature and definitions (e.g. the ICSD-3 [7], or Diagnostic and Statistical Manual of Mental Disorders, Fifth Edition [25]) and improvements in diagnostic testing, leading to better differentiation of NT2 from other sleep disorders.

The estimated point prevalence and incidence of NT1, NT2, and overall narcolepsy were higher in females than in males. This trend was consistent across all years analysed and with recently published studies utilizing healthcare claims data or registry data [9, 10, 12]. However, an older population analysis using data from 1960 to 1989 reported a higher prevalence of narcolepsy in males than females [13], whereas a study evaluating the prevalence of symptoms associated with narcolepsy found no difference [15]. Some studies suggest that females may experience longer delays to diagnosis and are more likely to be misdiagnosed [26], while the potential for underestimation of narcolepsy cases among males from claims data owing to documented reluctance of men to seek medical advice cannot be discounted [27].

Age-specific patterns in prevalence and incidence revealed distinct trends for NT1 and NT2. Among individuals with NT1, point prevalence was highest in adults aged 25–44 years, whereas NT2 prevalence peaked in adults aged 35–54 years. Notably, the ratio of NT2 to NT1 prevalence increased substantially with older age, from 2.0- to 2.4-fold higher among individuals with narcolepsy under the age of 20 years, 3.7-fold higher for NT2 versus NT1 among individuals aged 45–54 years, and 5.3-fold higher for NT2 versus NT1 among those aged 65 years and older. These findings suggest that NT2 may be disproportionately more common, or more frequently diagnosed, in older adults compared with NT1. The increasing presence of comorbidities in older adults that results in excessive daytime sleepiness and reduction in cataplexy frequency with age may also increase NT2 incidence [28], especially in the United States where the testing protocols, including CSF orexin measurement, are not frequently utilized. Previous studies have suggested the frequency and severity of cataplexy episodes peak during adolescence and early adulthood, and that over time, cataplexy attacks often become less frequent with increasing age, although the reason is unknown [29–31]. This change in symptom frequency potentially results in older individuals with underlying NT1 receiving NT2 billing codes following the stabilization of excessive daytime sleepiness and cataplexy symptoms. Consistent with the prevalence trends, incidence rates for both NT1 and NT2 were highest among adults aged 25–44 years, indicating that initial disease recognition may be concentrated in early to mid-adulthood.

A few limitations of the study should be considered when interpreting these results. The validation of the narcolepsy case definitions relied on chronological listings of structured EMR data, available laboratory data, and claims data, which may not fully align with clinically confirmed diagnoses based on sleep tests. In particular, diagnostic elements from the ICSD-3-TR, including results from MSLT and nocturnal polysomnography tests [7], were not available in the structured EMR data. Although sleep tests play an essential role in differentiating narcolepsy from other non-recurrent sleep disorders, such tests are typically performed only once for each individual at the time of diagnosis, and insufficient data from within the study period were available for inclusion in the case definition. The study was additionally limited by the number of years of enrollment in the database. Cases that occurred during a patient’s medical history before their enrollment in the insurance plan were not captured, which may have resulted in missed prevalent cases as well as misclassification of prevalent cases as incident cases. Finally, this study estimated diagnosed prevalence only and did not assess undiagnosed cases in the population at large, which is estimated to be as much as half of all diagnosed people [2, 32].

Nonetheless, this study has a number of key strengths. To our knowledge, this is the first study to validate claims-based case definitions of narcolepsy to reduce the potential for misclassification in the estimation of point prevalence and annual incidence rates. Furthermore, the participant profiles were comprehensive in nature and included a chronological listing of structured EMR data, available laboratory test data, and claims data, including all diagnosis codes, procedure codes, and medication codes and the provider specialty associated with each code. Another strength is that practicing sleep specialists completed the adjudication to quantify potential misclassification of diagnoses and provide more credible prevalence and incidence estimates. An additional strength of the study was the generalizability of the results to the US population through the inclusion of individuals with commercial, Medicaid, and Medicare Advantage insurance coverage. The participant distribution in the prevalence study population ensured representation across a broad spectrum of individuals, including employees and their dependents, low-income groups, and adults aged 65 years or older.

## Conclusion

This study provides refined estimates of the diagnosed prevalence and incidence of NT1, NT2, and overall narcolepsy in the United States, using validated clinician-adjudicated case definitions to enhance diagnostic accuracy. The data demonstrate that the diagnosed prevalence of NT1 is relatively low (<200 000 cases) in the United States, while NT2 emerged as the predominant subtype, with prevalence over 3 times that of NT1. Although NT1 incidence remained stable from 2020 to 2023, NT2 and overall narcolepsy incidence declined, potentially reflecting shifts in diagnostic practices or healthcare access. Age- and sex-related patterns were evident, with NT1 and NT2 peaking in different age groups and higher rates observed in females across all definitions. These findings offer critical insights to inform future research, as this is the first epidemiologic study in narcolepsy to mitigate the risk of case misclassification inherent to claims-based research, providing more reliable and precise estimates of narcolepsy prevalence and incidence than previously reported.

## Supplementary Material

zpag043_HV_manuscript_US_ODD_Suppl_09Apr2026

## Data Availability

Sharing of data from HealthVerity databases is not permitted under the usage license. Access to the dataset is available for purchase from HealthVerity (Contact: https://healthverity.com/data-ecosystem-partners/).
