# Supplementary material for: The prevalence and incidence of narcolepsy in the United States: a real-world observational study using a validated narcolepsy case definition
Source: Sleep Adv. 2026 Apr 17;7(2):zpag043. doi: 10.1093/sleepadvances/zpag043 (PMC13156510; doi:10.1093/sleepadvances/zpag043)
Supplement: zpag043_HV_manuscript_US_ODD_Suppl_09Apr2026 [file zpag043_hv_manuscript_us_odd_suppl_09apr2026.docx]

# The prevalence and incidence of narcolepsy in the United States: a real-world observational study using a validated narcolepsy case definition

Somraj Ghosh^1^, Veena Hoffman^2^, Brian Calingaert^2^, David T. Plante^3^, Lois E. Krahn^4^, Alice Cai^1^, Mary S. Anthony^2,†^, Shreya Dave^1^, Satish Rao^1^ and Stephen Crawford^1,^*

^1^Takeda Development Center Americas, Inc., Cambridge, MA, United States, ^2^RTI Health Solutions, Research Triangle Park, NC, United States, ^3^University of Wisconsin School of Medicine and Public Health, Madison, WI, United States, ^4^Mayo Clinic College of Medicine, Phoenix, AZ, United States

*Corresponding author. Stephen Crawford, Address: 500 Kendall Street, Cambridge, MA 02142, United States. Email: [stephen.crawford@takeda.com](mailto:stephen.crawford@takeda.com).

^†^Dr Anthony sadly passed away in December 2025 after submission of the article. As an employee of RTI Health Solutions her substantial contributions to this research and paper warrant posthumous authorship.

**SUPPLEMENTARY MATERIAL**

**Descriptions of data sources**

**Supplementary Table S1.** Claims-based operational definitions of NT1, NT2, and overall narcolepsy

**Supplementary Table S2.** Study population demographic characteristics for estimation of point prevalence on December 31, 2023, and annual incidence rate during 2023

**Supplementary Table S3.** Unadjusted and PPV-adjusted annual incidence rates per 100 000 person-years for NT1, NT2, and overall narcolepsy: 2020 to 2023

**Supplementary Figure S1.** Unadjusted annual incidence of NT1, NT2, and overall narcolepsy by sex and age during 2023, with varying NT2:NT1 ratio with age

**Descriptions of data sources**

The HealthVerity (HV) Inovalon closed claims database includes deidentified data from approximately 166 million individuals with coverage through commercial health, Medicaid, or Medicare Advantage plans. A subset of approximately 48 million individuals with closed claims linked to their electronic medical records (EMRs) was leveraged to validate the operational definitions used in this study.

The HV Veradigm EMR data provide information relating to encounters with medical professionals, for which the provider records detail in an electronic version of the individuals’ chart, including vital statistics, individual history, and laboratory test orders and results. The EMR data have been available since January 1990 and include data for >174 million individuals, with a unique identifier to link encounter data over time and across providers. The data are sourced from over 20 000 healthcare practices across the United States using several different EMR systems.

Source 42 is a multispecialty EMR vendor that includes clinical records since 2003 from both ambulatory and specialty practices for nearly 60 million unique individuals. Linkage between the claims and EMR data sources was conducted by HV through a Health Insurance Portability and Accountability Act–compliant probabilistic matching and tokenization process. Data recorded from March 31, 2018, to December 31, 2023, in the HV ecosystem of databases were included in the analysis.

**Supplementary Table S1.** Claims-based operational definitions of NT1, NT2, and overall narcolepsy

| Indication | Claims-based operational definition |
| --- | --- |
| NT1 (subset of the overall narcolepsy definition) | - ≥2 medical claims with recorded diagnosis codes for NT1 or NT2 ≥60 and ≤548 days apart AND the most recent narcolepsy medical claim had a recorded diagnosis code for NT1 but not NT2.^a^   OR   - A medical claim with a recorded NT1 or NT2 diagnosis code and a claim with a prescription claim for pitolisant, sodium oxybate, or low sodium/mixed salt oxybate on the diagnosis date or within 365 days after the diagnosis date AND the most recent medical claim for narcolepsy had a recorded diagnosis code for NT1 but not NT2.^a^ Individuals with a medical claim with a recorded diagnosis code for idiopathic hypersomnia (ICD-10-CM G47.11 or G47.12) +/− 180 days of a prescription claim for pitolisant, sodium oxybate, or low sodium/mixed salt oxybate were excluded.   OR   - A procedure claim for an MSLT and a medical claim with a recorded NT1 or NT2 diagnosis code within 365 days after (not inclusive of) the date of the procedure claim AND the most recent medical claim for narcolepsy had a recorded diagnosis code for NT1 but not NT2.^a^ |
| NT2 (subset of the overall narcolepsy definition) | - ≥2 medical claims with recorded diagnosis codes for NT1 or NT2 ≥60 and ≤548 days apart AND the most recent narcolepsy medical claim had a recorded diagnosis code for NT2 but not NT1.^a^   OR   - A medical claim with a recorded NT1 or NT2 diagnosis code and a claim with a prescription claim for pitolisant, sodium oxybate, or low sodium/mixed salt oxybate on the diagnosis date or within 365 days after the diagnosis date AND the most recent medical claim for narcolepsy had a recorded diagnosis code for NT2 but not NT1.^a^ Individuals with a medical claim with a recorded diagnosis code for idiopathic hypersomnia (ICD-10-CM G47.11 or G47.12) +/− 180 days of a prescription claim for pitolisant, sodium oxybate, or low sodium/mixed salt oxybate were excluded.   OR   - A procedure claim for an MSLT and a medical claim with a recorded NT1 or NT2 diagnosis code within 365 days after (not inclusive of) the date of the procedure claim AND the most recent medical claim for narcolepsy had a recorded diagnosis code for NT2 but not NT1.^a^ |
| Overall narcolepsy | - ≥2 medical claims with recorded diagnosis codes for NT1 or NT2 (ICD-10-CM G47.411 or G47.419 or G47.421 or G47.429) ≥60 and ≤548 days apart.   OR   - A medical claim with a recorded NT1 or NT2 diagnosis code and a claim with a prescription claim for pitolisant, sodium oxybate, or low sodium/mixed salt oxybate within 365 days after the NT1 or NT2 diagnosis code (individuals with a prescription claim and diagnosis code on the same day were included). Individuals with a medical claim with a recorded diagnosis code for idiopathic hypersomnia (ICD-10-CM G47.11 or G47.12) +/− 180 days of a prescription claim for pitolisant, sodium oxybate, or low sodium/mixed salt oxybate were excluded.   OR   - A procedure claim for an MSLT and a medical claim with a recorded NT1 or NT2 diagnosis code within 365 days after (not inclusive of) the date of the procedure claim. |

^a^Use of the most recent diagnosis code to classify individuals as having NT1 versus NT2 was to reflect possible clinical progression from NT2 to NT1 and/or the result of the diagnostic process.

Note: All timeframes listed here were for the purposes of identifying potential cases of NT1, NT2, or overall narcolepsy. However, all available information from March 1, 2018, through December 31, 2023, was included in the participant profiles for adjudication.

Abbreviations: ICD-10-CM, International Classification of Diseases, 10th Revision, Clinical Modification; MSLT, multiple sleep latency test; NT1, narcolepsy type 1; NT2, narcolepsy type 2.

**Supplementary Table S2.** Study population demographic characteristics for estimation of point prevalence on December 31, 2023, and annual incidence rate during 2023

|  | **Prevalence population (December 31, 2023)** | | **Incidence population (2023)** | |
| --- | --- | --- | --- | --- |
|  | **Denominator population** | **Narcolepsy  prevalent cases** | **Denominator population** | **Narcolepsy  incident cases** |
| Study population |  |  |  |  |
| Total, *N* (%) | 70 548 164 (100) | 47 296 (100) | 62 809 285 (100) | 5831 (100) |
| Sex, *n* (%) |  |  |  |  |
| Female | 37 560 228 (53.2) | 31 200 (66.0) | 33 557 992 (53.4) | 3853 (66.1) |
| Male | 32 987 936 (46.8) | 16 096 (34.0) | 29 251 293 (46.6) | 1978 (33.9) |
| Age, years |  |  |  |  |
| Mean (SD) | 35.2 (20.7) | 39.1 (15.9) | 35.3 (20.9) | 39.8 (16.8) |
| Median (IQR) | 33 (17–52) | 38 (27–51) | 33 (17–53) | 39 (26–52) |
| Age group, years, *n* (%) |  |  |  |  |
| <5 | 1 526 039 (2.2) | 26 (0.1) | 884 978 (1.4) | 1 (0.0) |
| 5–9 | 5 905 776 (8.4) | 425 (0.9) | 5 420 133 (8.6) | 39 (0.7) |
| 10–14 | 6 650 384 (9.4) | 1678 (3.5) | 6 252 639 (10.0) | 163 (2.8) |
| 15–19 | 6 905 576 (9.8) | 3635 (7.7) | 6 525 045 (10.4) | 519 (8.9) |
| 20–24 | 5 723 836 (8.1) | 3784 (8.0) | 5 168 350 (8.2) | 548 (9.4) |
| 25–34 | 10 070 873 (14.3) | 10 112 (21.4) | 8 495 338 (13.5) | 1135 (19.5) |
| 35–44 | 9 511 236 (13.5) | 10 343 (21.9) | 8 233 134 (13.1) | 1194 (20.5) |
| 45–54 | 8 900 967 (12.6) | 8633 (18.3) | 7 816 735 (12.4) | 974 (16.7) |
| 55–59 | 4 418 244 (6.3) | 3570 (7.5) | 3 916 539 (6.2) | 453 (7.8) |
| 60–64 | 4 683 085 (6.6) | 2384 (5.0) | 4 209 396 (6.7) | 395 (6.8) |
| 65+ | 6 252 148 (8.9) | 2706 (5.7) | 5 886 998 (9.4) | 410 (7.0) |
| Insurance type, *n* (%) |  |  |  |  |
| Commercial | 31 415 592 (44.5) | 24 438 (53.8) | 27 041 303 (43.1) | 3095 (53.1) |
| Medicaid | 34 588 961 (49.0) | 17 622 (37.3) | 31 719 324 (50.5) | 2387 (40.9) |
| Medicare Advantage | 3 484 236 (4.9) | 2578 (5.5) | 3 021 138 (4.8) | 289 (5.0) |
| Unknown | 1 059 375 (1.5) | 1658 (3.5) | 1 027 520 (1.6) | 60 (1.0) |

Abbreviations: IQR, interquartile range; SD, standard deviation.

**Supplementary Table S3.** Unadjusted and PPV-adjusted annual incidence rates per 100 000 person-years for NT1, NT2, and overall narcolepsy: 2020 to 2023

| **Calendar year** | **NT1** | |  | **NT2** | |  | **Overall narcolepsy** | |
| --- | --- | --- | --- | --- | --- | --- | --- | --- |
|  | **Unadjusted** | **Adjusted (95% CI)** |  | **Unadjusted** | **Adjusted (95% CI)** |  | **Unadjusted** | **Adjusted^a^ (95% CI)** |
| 2020 | 2.1 | 1.7 (1.5–1.8) |  | 9.1 | 7.6 (6.8–8.3) |  | 11.3 | 9.0 (8.0–9.8) |
| 2021 | 2.3 | 1.9 (1.7–2.0) |  | 8.6 | 7.2 (6.5–7.8) |  | 11.0 | 8.8 (7.8–9.6) |
| 2022 | 2.1 | 1.7 (1.5–1.8) |  | 8.3 | 7.0 (6.2–7.6) |  | 10.5 | 8.4 (7.5–9.1) |
| 2023 | 2.0 | 1.6 (1.4–1.8) |  | 7.6 | 6.4 (5.7–6.9) |  | 9.6 | 7.7 (6.8–8.4) |

Abbreviations: CI, confidence interval; NT1, narcolepsy type 1; NT2, narcolepsy type 2; PPV, positive predictive value.

**Supplementary Figure S1.** Unadjusted annual incidence of narcolepsy type 1 (NT1), narcolepsy type 2 (NT2), and overall narcolepsy by sex and age during 2023, with varying NT2:NT1 ratio with age. CI, confidence interval.

**
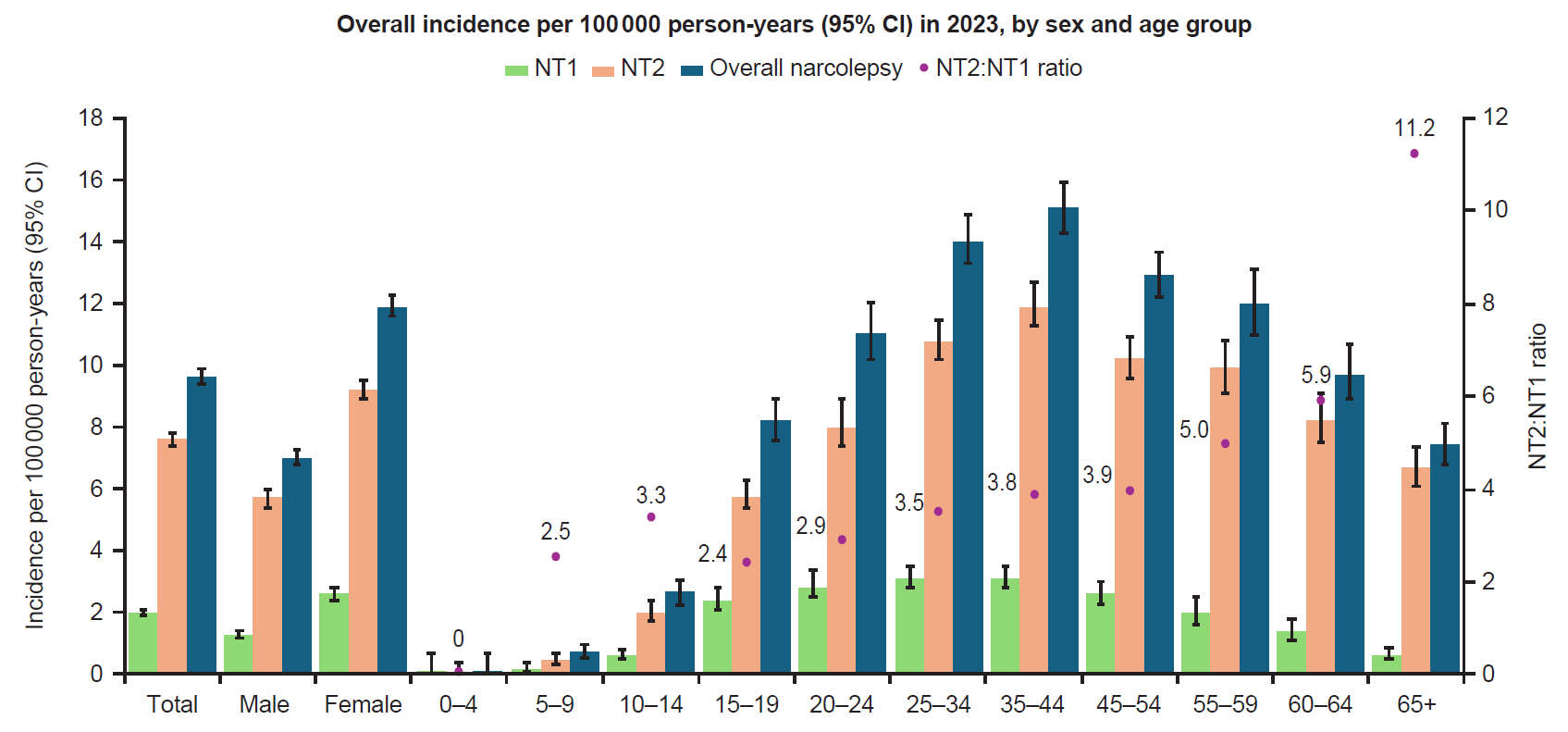
**
